# Genomic analysis of methicillin-resistant *Staphylococcus aureus* clonal complex 239 isolated from Danish patients with and without an international travel history

**DOI:** 10.3389/fmicb.2022.1016829

**Published:** 2022-11-24

**Authors:** Jasmine Coppens, Basil Britto Xavier, Jelle Vlaeminck, Jesper Larsen, Christine Lammens, Sandra Van Puyvelde, Herman Goossens, Anders Rhod Larsen, Surbhi Malhotra-Kumar

**Affiliations:** ^1^Laboratory of Medical Microbiology, Vaccine and Infectious Disease Institute, University of Antwerp, Antwerp, Belgium; ^2^Department of Bacteria, Parasites and Fungi, Statens Serum Institut, Copenhagen, Denmark; ^3^Cambridge Institute of Therapeutic Immunology and Infectious Disease, University of Cambridge School of Clinical Medicine, Cambridge Biomedical Campus, Cambridge, United Kingdom

**Keywords:** MRSA, CC239, ST239, *sasX*, Denmark, WGS

## Abstract

**Introduction:**

International travel has been a major determinant for the introduction of pathogens such as methicillin-resistant *Staphylococcus aureus* (MRSA) into naïve geographic areas. MRSA clonal complex 239 (CC239) is a highly virulent clone that is predominant in Asia. The objective of this study was to determine the geographic origin of MRSA CC239 isolates recovered from Danish cases with or without a history of international travel during 2004–2016.

**Materials and methods:**

Human MRSA isolates with *spa* types t030 and t037 (*n* = 60) were obtained from the National Reference Laboratory for Antimicrobial Resistance. For each case, the following data were collected from notification forms: sex, age, isolation year, specimen source (screening swab or clinical sample), infection type, and international travel history. All isolates were whole-genome sequenced, and a comparative genome and phylogenetic analysis was performed.

**Results:**

The majority of isolates originated from skin and soft tissue (SST) infections and screening swabs. In 31 out of 60 cases reported international travel to different parts of the world. Fifty-four isolates belonged to CC239, including sequence type 239 (ST239) (*n* = 43), ST241 (*n* = 5), ST4377 (*n* = 2), ST4378 (*n* = 1), ST1465 (*n* = 1), ST343 (*n* = 1), and ST592 (*n* = 1). The majority of the CC239 MRSA isolates (40/54) belonged to well-known geographic clades, including the Asian (*n* = 12), Serbian (*n* = 11), South American (*n* = 2), and Turkish (*n* = 15). Most MRSA ST239 isolates belonging to the highly virulent Asian clade carried *sasX* and were recovered from individuals who had travelled to Asia, Africa and the Middle East.

**Conclusion:**

Our data reveal multiple introductions of MRSA CC239 into Denmark through international travel, which highlights the importance of continued genomic surveillance of MRSA in persons returning from international travel to areas where MRSA is endemic.

## Introduction

Methicillin-resistant *Staphylococcus aureus* (MRSA) is a major cause of difficult-to-treat infections around the world. Some MRSA clones have evolved into multiple clades which can be distinguished by core genome single nucleotide polymorphisms (SNPs) and are correlated with geographical origin (Lee et al., 2018). In Northern Europe, especially in Scandinavian countries, infections due to MRSA are relatively rare, accounting for ~2% of all *S*. *aureus* bloodstream infections (One Health Trust, 2022), but in recent times a steady increase in MRSA infections has been noted Danish Programme for surveillance of antimicrobial consumption and resistance in bacteria from food animals, food and humans DANMAP 2021.^1^ In 2020, ~17% (215/1281) of all Danish patients with MRSA infection had likely acquired MRSA outside Denmark, supporting that international travel is a risk factor for the introduction of MRSA into Denmark (Larsen et al., 2008a; Zhou et al., 2014). A longitudinal study from Sweden showed a ~ 10-fold rise in MRSA cases between 2000 and 2010, of which ~76% of the infecting MRSA isolates originated from outside Sweden (Larsson et al., 2014). The most common route of acquisition is through direct skin contact with colonized individuals, fomites or animals during an overseas trip (Zhou et al., 2014).

MRSA clonal complex 239 (CC239) comprises the sequence type 239 (ST239) clone, which is widespread globally (Oliveira et al., 1998; Ko et al., 2005; Harris et al., 2010; Li et al., 2011). Several ST239 variants have been previously described, including the Brazilian, Hungarian, Portuguese, Viennese, Indian, Asian, and Eurasian clades (Harris et al., 2010; Wang et al., 2014; Monecke et al., 2018; De Backer et al., 2019). ST239 is endemic in Southeast Asia where it causes more than 70% of hospital-acquired MRSA infections (Ko et al., 2005). Asian ST239 clade isolates are highly virulent and harbour different virulence factors, such as the *sasX* gene present on a φSPβ-like prophage and encoding a virulence factor involved in colonization and immune evasion of the bacterium (Wang et al., 2014; Nakaminami et al., 2017; De Backer et al., 2019), which might have facilitated its pandemic spread across Asia.

Here, we investigated the population structure and origin of MRSA CC239 isolates recovered from 60 Danish cases with or without a history of international travel during 2004–2016.

## Materials and methods

### Clinical information and study isolates

The study population included all Danish cases who tested positive for MRSA with the CC239-associated *spa* types t030 or t037 during 2004–2016. For each case, the following data were collected from notification forms: sex, age, isolation year, specimen source (screening swab or clinical sample), infection type, and international travel history 12 months prior to diagnosis. Detailed data on the characteristics of MRSA isolates were extracted from the national MRSA database. The regional clinical microbiology laboratories performed *S*. *aureus* identification and established methicillin resistance by use of standard laboratory methods and forwarded the isolates to the National Reference Laboratory for Antimicrobial Resistance at Statens Serum Institut, where MRSA identification was confirmed by PCR detection of *mecA* and *spa* typing (Larsen et al., 2008b). Collection and use of data were approved by the Danish Data Protection Agency (protocol no. 2001-14-0021).

### Genotypic characterization

Multilocus sequence typing (MLST) was performed as previously described (Enright et al., 2000). STs and CCs were assigned through the *Staphylococcus aureus* PubMLST database. All isolates were tested for the presence of *sasX* by PCR as described previously (Li et al., 2012). Positive isolates were further investigated for the presence of the φSPβ-like prophage by PCR and Sanger sequencing as described elsewhere (De Backer et al., 2019).

### Whole-genome sequencing and bioinformatics analyses

All isolates were subjected to DNA extraction using the Master Pure Complete DNA and RNA purification kit (Epicentre, Madison, WI, United States), according to the manufacturer’s protocol. We used the Illumina Nextera XT kit for library construction and paired-end reads of 250 bp each were generated on a Miseq platform (Illumina Inc., San Diego, United States). The raw sequences were analyzed using the bacterial whole-genome sequencing analysis pipeline Bacpipe v.1.2 (Xavier et al., 2020). The online server^2^ was used to identify prophages (Arndt et al., 2016). To study the genetic relatedness of isolates, we have utilized gene-by-gene approach using chewBBACA (Silva et al., 2018). we generated a study-specific ST239 scheme based on 27 publicly available MRSA ST239 genomes representing each geographic clades (Supplementary Table S1). Phylogenetic relationship was visualized using iTOL (Letunic and Bork, 2019), and geographic clades were defined based on published MRSA ST239 isolates from (Harris et al., 2010; De Backer et al., 2019). All sequence data from this study have been deposited under BioProject ID: PRJNA674016.

## Results

### Clinical information

**Figure 1:**
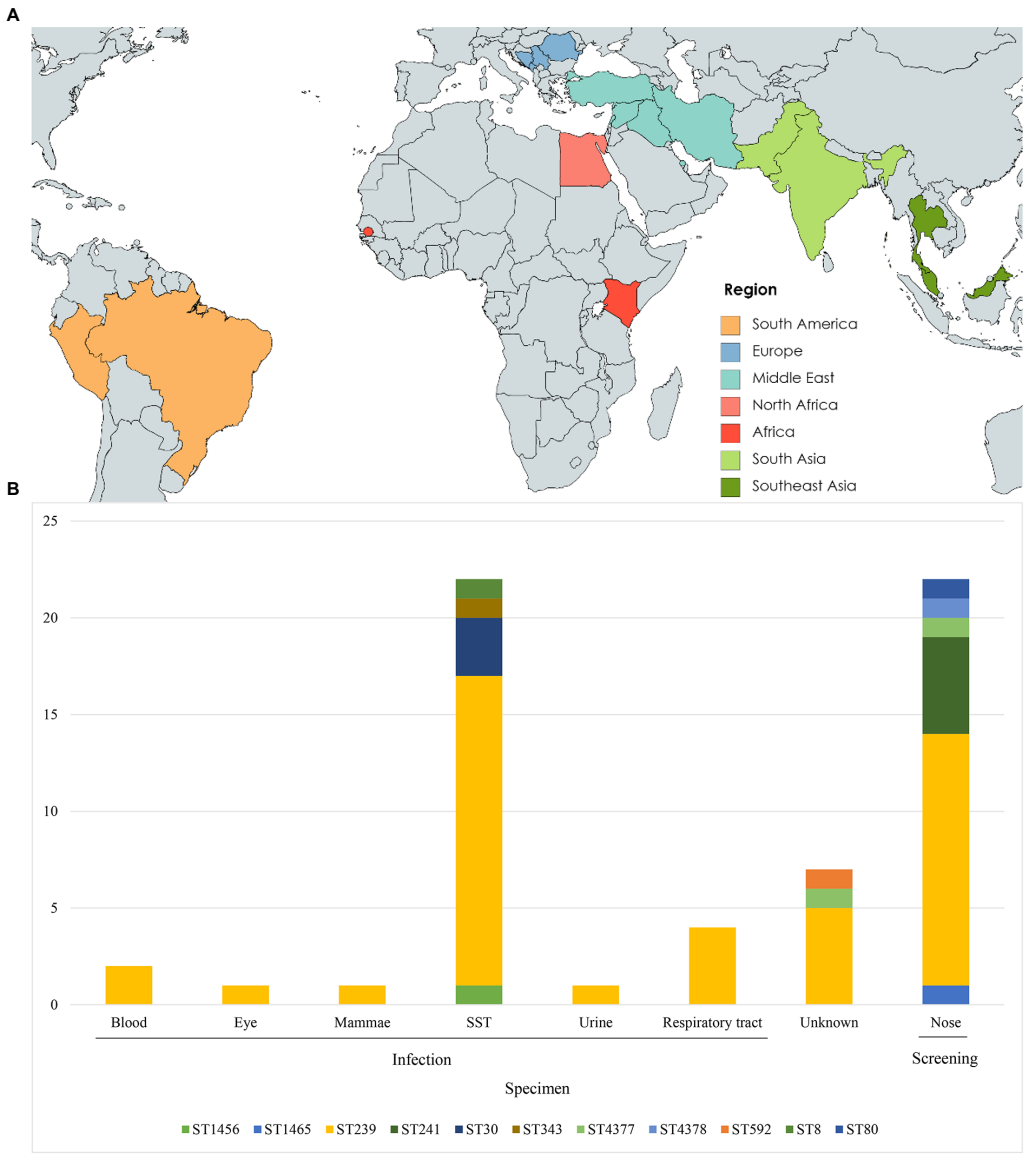
Geographic origin and sequence type (ST) distribution **(A)** Travel destinations of individuals (*n* = 31) entering Denmark used in this study. Colors represent geographic regions of travel destinations. **(B)** ST type distribution of isolates (*n* = 60) by specimen type. SST, skin and soft tissue.

The 60 isolates originated from SST (*n* = 22), respiratory tract (*n* = 4), blood (*n* = 2), urine (*n* = 1), mammary gland (*n* = 1), eye (*n* = 1), screening swabs (*n* = 22), and unknown source (*n* = 7) (Table 1). Of the 60 cases, 31 had a history of travel to Balkan (*n* = 5), Africa (*n* = 3), North Africa (*n* = 2), South America (*n* = 2), South Asia (*n* = 4), Southeast Asia (*n* = 3) and the Middle East (*n* = 12), whereas 26 had no history of international travel. Three cases had unknown travel histories (Figures 1A,B).

**Table 1:** Clinical information and characteristics of isolates used in this study.

Strain ID	Clinical information		Isolate information				
	Isolation year	Specimen^b^	Travel	Region	*spa* type	ST	CC	CC239 clade ^c^	*sasX*
56018	2007	Respiratory tract	No travel	No travel		t037	ST239	CC239	Asian	−
71855	2010	Blood	Malaysia	Southeast Asia		t037	ST239	CC239	Asian	+
56967	2007	Eye	No travel	No travel		t037	ST239	CC239	Asian	+
61133	2008	SST	No travel	No travel		t037	ST239	CC239	Asian	+
61134	2008	SST	No travel	No travel		t037	ST239	CC239	Asian	+
71679	2010	SST	No travel	No travel		t037	ST239	CC239	Asian	+
71971	2010	SST	No travel	No travel		t037	ST239	CC239	Asian	+
103052	2015	SST	Iran	Middle East		t037	ST239	CC239	Asian	+
71927	2010	Unknown	No travel	No travel		t037	ST239	CC239	Asian	+
56389	2007	Screening	Gambia	Africa		t037	ST239	CC239	Asian	+
81894	2011	Screening	Iran	Middle East		t037	ST239	CC239	Asian	+
719687	2010	Screening	No travel	No travel		t037	ST239	CC239	Asian	+
89102	2013	Respiratory tract	No travel	No travel		t037	ST239	CC239	Serbian	−
89110	2013	Respiratory tract	No travel	No travel		t037	ST239	CC239	Serbian	−
78938	2011	SST	Lebanon	Middle East		t037	ST239	CC239	Serbian	−
91354	2014	SST	No travel	No travel		t037	ST239	CC239	Serbian	−
82200	2012	SST	Serbia/Montenegro	Europe		t037	ST239	CC239	Serbian	−
109880	2016	SST	Syria	Middle East		t037	ST239	CC239	Serbian	−
109478	2016	Screening	Bosnia and Herzegovina	Europe		t037	ST239	CC239	Serbian	−
68078	2009	Screening	Egypt	North Africa		t037	ST239	CC239	Serbian	−
78770	2011	Screening	Lebanon	Middle East		t037	ST239	CC239	Serbian	−
107673	2016	Screening	Serbia/Montenegro	Europe		t037	ST239	CC239	Serbian	−
106227	2016	Screening	Thailand	Southeast Asia		t037	ST239	CC239	Serbian	−
56429	2007	Mammary gland	Peru	South America		t037	ST239	CC239	South America	−
75633	2011	SST	Brazil	South America		t037	ST239	CC239	South America	−
61001	2008	Blood	No travel	No travel		t030	ST239	CC239	Turkish	−
89269	2013	Respiratory tract	Turkey	Middle East		t030	ST239	CC239	Turkish	−
85593	2013	SST	No travel	No travel		t030	ST239	CC239	Turkish	−
108285	2016	SST	India	South Asia		t030	ST239	CC239	Turkish	−
99359	2015	SST	Iran	Middle East		t030	ST239	CC239	Turkish	−
57664	2007	SST	Pakistan	South Asia		t030	ST239	CC239	Turkish	−
100665	2016	SST	Romania	Europe		t030	ST239	CC239	Turkish	−
92214	2014	Unknown	India	South Asia		t030	ST239	CC239	Turkish	−
56427	2007	Unknown	Romania	Europe		t030	ST239	CC239	Turkish	−
45137	2005	Unknown	Unknown	Unknown		t030	ST239	CC239	Turkish	−
110614	2016	Unknown	Unknown	Unknown		t030	ST239	CC239	Turkish	−
110480	2016	Urine	No travel	No travel		t030	ST239	CC239	Turkish	−
89448	2013	Screening	No travel	No travel		t030	ST239	CC239	Turkish	−
27790	2009	Screening	Pakistan	South Asia		t030	ST239	CC239	Turkish	−
66910	2009	Screening	Turkey	Middle East		t030	ST239	CC239	Turkish	−
83627	2012	SST	No travel	No travel		t037	ST239	CC239	NA	−
73198	2010	Screening	Iraq	Middle East		t037	ST239	CC239	NA	−
100940	2015	Screening	Lebanon	Middle East		t037	ST239	CC239	NA	+
55017	2007	SST	Thailand	Southeast Asia		t037	ST343	CC239	ND	+
55526	2007	Unknown	No travel	No travel		t037	ST4377	CC239	ND	−
40836	2004	Unknown	Unknown	Unknown		t037	ST592	CC239	ND	+
70060	2010	Screening	Bahrain	Middle East		t037	ST241	CC239	ND	−
57193	2007	Screening	Iran	Middle East		t030	ST1465	CC239	ND	−
55461	2007	Screening	Egypt	North Africa		t037	ST241	CC239	ND	+
86496	2013	Screening	Kenya	Africa		t037	ST241	CC239	ND	+
103046	2015	Screening	Kenya	Africa		t037	ST241	CC239	ND	+
56193	2007	Screening	No travel	No travel		t037	ST241	CC239	ND	+
56835	2007	Screening	No travel	No travel		t037	ST4377	CC239	ND	+
57963	2007	Screening	No travel	No travel		t037	ST4378	CC239	ND	+
57959	2007	Screening	No travel	No travel		t037	ST80	CC80	ND	−
57401	2007	SST	No travel	No travel		t030	ST1456	CC30	ND	−
57891	2007	SST	No travel	No travel		t030	ST8	CC80	ND	−
61875	2008	SST	No travel	No travel		t037	ST30	CC30	ND	−
61908	2008	SST	No travel	No travel		t037	ST30	CC30	ND	−
82259	2012	SST	No travel	No travel		t037	ST30	CC30	ND	−

### Population structure

Of the 60 MRSA isolates, 54 belonged to STs within CC239, including ST239 (*n* = 43), ST241 (*n* = 5), ST343 (*n* = 1), ST592 (*n* = 1), ST1465 (*n* = 1), ST4377 (*n* = 2), and ST4378 (*n* = 1) (Table 1). The remaining isolates belonged to ST30 (*n* = 3) and ST1456 (*n* = 1) within CC30, and to ST8 within CC8 (*n* = 1) and ST80 (*n* = 1) within CC80.

### Population structure of MRSA ST239

**Figure 2:**
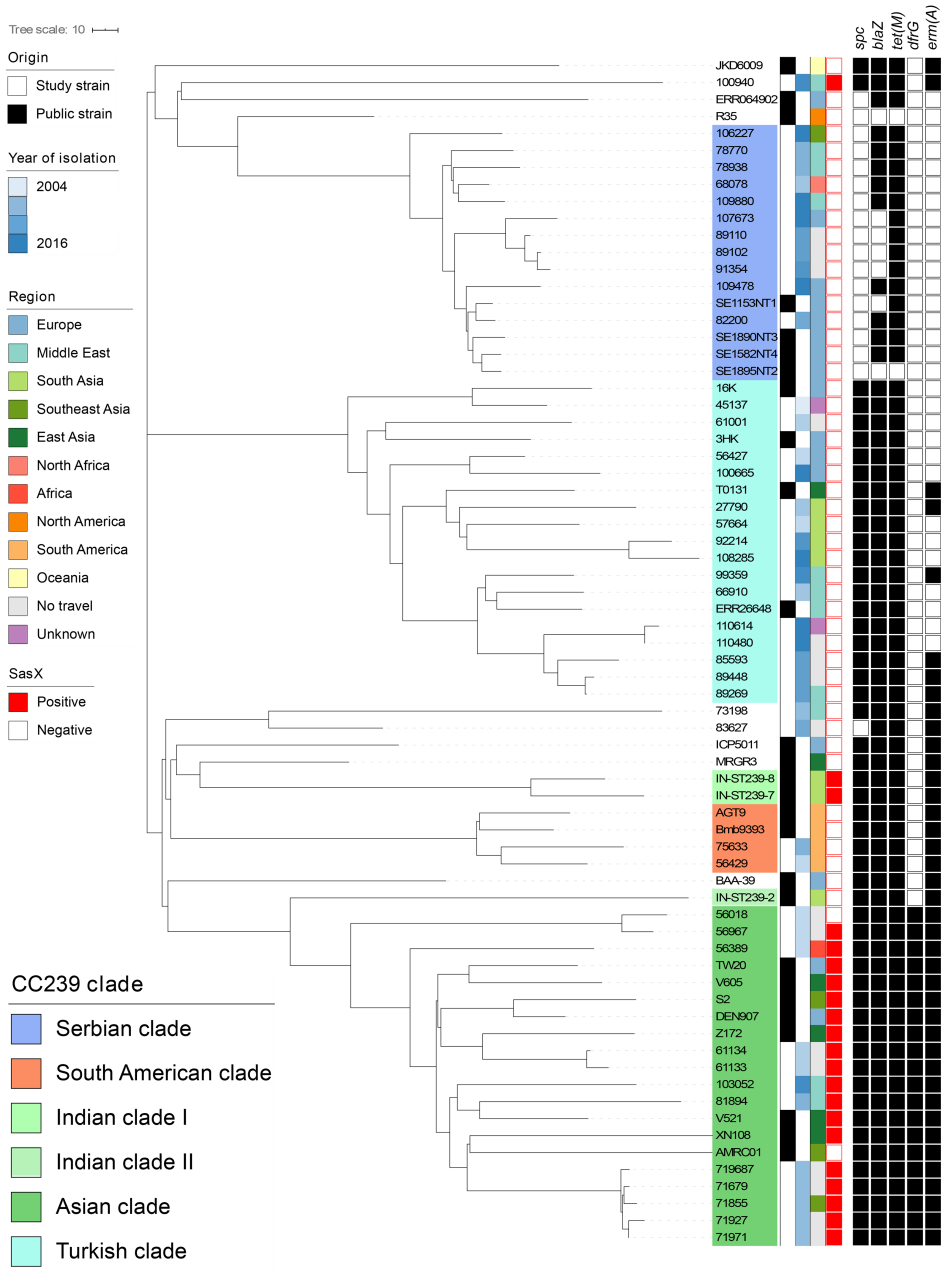
Core-genome phylogeny of all MRSA ST239 isolates isolated from Denmark (*n* = 40) and published context sequences (*n* = 27). Clustering is done in line with Harris et al. (2010) and De Backer et al. (2019) clades are indicated by colored overlays. Metadata is visualized in the lanes in compliance to the legend, from left to right: (i) Origin of the isolate; (ii) Year of isolation; (iii) Region of travel; (iv) *sasX* carriage; and (v) Carriage of antimicrobial resistance genes *spc*, *blaZ*, *tet(M)*, *dfrG*, and *erm(A)* with black signifying carriage and white non-carriage. The context sequences were downloaded from Genbank and Sanger Institute. Branch lengths represent the substitution rate per position in the core-genome alignment as indicated in the scale bar. The tree is publicly available on MicroReact (https://microreact.org/project/oxRfLg4vYw4KqeXRdUXb33).

Allelic-loci differences were calculated among the 43 MRSA ST239 genomes from this study and the collection of 27 publicly available MRSA ST239 genomes, and a core-genome phylogeny was constructed (Figure 2). In total, 3,123 loci were determined, from which 921 accessory-genome loci were removed and 2,161 core-genome loci were used to calculate the differences between the strains. The MRSA ST239 isolates were assigned to the Turkish (*n* = 15), Asian (*n* = 12), Serbian (*n* = 11) and South American (*n* = 2) clades (Figure 2). Three isolates could not be assigned to a clade.

MRSA ST239 isolates belonging to the Asian clade clustered closely with published Asian clade isolates (S2, Z172, DEN907, TW20, V605, AMRC01, XN108, and V521). Isolates from the same country and different years were closely related, for instance isolates originating from Kenya and Iran, respectively (Figure 2). Interestingly, several isolates obtained from patients with no international travel history (*n* = 8) belonged to the Asian clade, and these clustered according to the isolation year (Figure 2). *N*one of the study isolates were closely related to DEN907 (Figure 2), which is a known Asian clade reference strain isolated in Denmark from a Thai patient (Harris et al., 2010).

*Additionally*, 15 isolates from individuals who reported travelling to Turkey (*n* = 2), Iran (*n* = 1), India (*n* = 2), Pakistan (*n* = 2), and Romania (*n* = 2), no international travel history (*n* = 4) and unknown (*n* = 2) clustered in the Turkish clade (Figure 2). Interestingly, *Turkish clade isolates obtained from cases* who reported travel *to Pakistan and India formed a distinct substructure*, *showing higher genetic relatedness to the Turkish clade reference strain*, *T0131 from China*, *than the TUR-1 isolated from Turkey*.

*Isolates from cases* who reported *travel to* Serbia (*n* = 2), *Lebanon (n = 2)*, Thailand (*n* = 1), Bosnia Herzegovina (*n* = 1), Syria (*n* = 1), Egypt (*n* = 1) and no travel (*n* = 3) clustered within the Serbian clade (Figure 2). These were closely related to Serbian isolates (SE1153NT1, SE1895NT2, SE1890NT3, SE1582NT2). Three monophyletic substructures were observed within the Serbian clade grouping non-European isolates separately from European and no travel isolates. *The study isolates from cases* who reported *travel to Brazil and Peru clustered according to their geographical origin*, *namely with the* South American clade reference strains BMB9393 and AGT9 *(*Figure 2*)*.

We found that the *sasX* gene was present in 11 out of 12 MRSA ST239 isolates from Asia, but not any other ST239 clade isolates (*n* = 28) (Figure 2 and Table 1). In addition, *sasX* was present in eight of the 14 MRSA CC239 belonging to STs other than ST239 (Table 1).

### Accessory genomic content of the MRSA ST239 isolates

Within each clade, isolates showed similarities in their core (*n* = 2,161) and accessory (*n* = 962) allelic-loci. All isolates from the Asian cluster (*n* = 12) showed presence of antimicrobial resistance genes *spc*, *blaZ*, *tet(M)*, *dfrG* and *erm(A)*, encoding resistance to, respectively, aminoglycoside, beta-lactam, tetracycline, trimethoprim and macrolide antibiotics (Figure 2; Supplementary Table S2). Isolates in this clade harboured 1–3 plasmid replicons and multiple virulence genes, including *lukD*, *lukE*, *hlgB*, *sek* and *seq* (Supplementary Table S2). The *rep21* plasmid replicon was predominantly present, except for one isolate (81894) (Supplementary Table S2).

In contrast, the Serbian clade showed only presence of the *blaZ* (11/11) and *tet(M)* (7/11) antimicrobial resistance genes (Figure 2; Supplementary Table S2), and contained fewer virulence genes than isolates from the Asian clade, namely *lukD*, *lukE*, *hlgB*, *sek* and *seq* (Supplementary Table S2). Within the Serbian clade, plasmid presence was similar to the Asian clade with individual isolates carrying up to three different plasmid *rep* genes. *rep10* was the most prevalent *rep* type (8/11), followed by *rep7* (6/11) and *rep21* (4/11) (Supplementary Table S2).

Finally, isolates from the Turkish (*n* = 15) and South American (*n* = 2) clades harboured, on average, fewer plasmids replicons (0–2 *rep* types). All isolates carried *spc*, *blaZ* and *tet(M)* antimicrobial resistance genes and virulence genes *lukD*, *lukE*, *sek* and *seq* (Figure 2; Supplementary Table S2).

## Discussion

This study reveals multiple introductions of MRSA CC239 into Denmark, and a predominance of the highly virulent Asian clade having been introduced by travel to Asia but also to Africa and the Middle East. The finding of genetically similar isolates from cases without any recent travel history indicates either an underreporting of travel or the spread of imported MRSA in the community. This study highlights the distinct clustering identified among CC239 MRSA isolated from Danish patients with an international travel history during 2004–2016. In total, isolates in four geographically defined clades were identified, i.e., the Serbian, Turkish, Asian and South American clades defined by previous studies (Harris et al., 2010; De Backer et al., 2019). Plasmid replicon abundance was higher in the Asian and Serbian clades as compared to the Turkish and South American ones, but some virulence factors were found across all clades. The latter two clades shared the same antimicrobial resistance genes while the other two had distinct antimicrobial resistance gene patterns.

ST239 *S*. *aureus* is an atypical member of canonical CC8, as it has unique features that distinguish it from other members of the clonal complex. These features include the presence of the *arcC*-allele, as well as other genes that are not typically found in CC8 strains. The difference in allele frequencies between ST239 and other CC8 strains suggests that this strain may have experienced positive selection, which could be due to the adaptive advantages conferred by the acquired genes. Further research is needed to determine the precise role that these genes play in the ecology and pathogenesis of ST239. The analysis of clonal complex sub-groups revealed distinct genotypic signatures that were used to classify individual CCs. ST239 strains were classified as CC239 rather than CC8 (Al Laham et al., 2015; Monecke et al., 2018).

In this study, the Turkish (Eurasian) clade was revealed to be most dominant with isolates from cases who had travelled across Asia, Africa and the Middle East. Importation of CC239 from these regions into Scandinavia has been described before (Stenhem et al., 2010). Interestingly, CC239 was also isolated from non-traveling cases (*n* = 19) with the majority *(n = 15) being part of either the Asian*, *Turkish and Serbian clades*. *Our Asian clade isolates did not cluster closely with DEN907*, *the Asian clade reference from Denmark*, *supporting that isolates might have been imported from the individual’s country of travel*. *These findings also imply a need for careful interpretation of clades named after the region where they were originally identified*.

*We identified an Asian clade isolate from a case who reported travel to Africa (56389)*. In recent years, there have been reports of Asian clade ST239 isolates in Africa (Abdulgader et al., 2015; Founou et al., 2018). This is not surprising, given the increasing global interconnectedness of the world today. This finding also suggests that there could be more ST239 isolates in Africa than have been found to date. *This might be because whole-genome sequencing data of African MRSA is scarce and these isolates were not yet analyzed up to the clade level* (Abdulgader et al., 2015). *A recent study found ST239 to be prevalent in Egypt although whole genome sequencing was not performed making it difficult to assess the presence of the Asian clade in the African continent* (Abd El-Hamid et al., 2022).

Furthermore, we identified a substructure within the Turkish clade that appears to be associated with travel to Asia. These isolates, originating from cases who had travelled to India and Pakistan, clustered with T0131, originating from China, indicating that there might be a Turkish subclade of the clade prevalent in the Asian region (Wang et al., 2014; Liao et al., 2020). However, *the presence of isolates from cases without an international travel history within the Asian clade might reflect local transmission events in Denmark*.

*Our study identified sasX positive isolates outside the Asian clade*. *sasX* is a known marker of the Asian clade and highly prevalent in South and East Asia, while also present in countries with low ST239 prevalence such as Japan (Nakaminami et al., 2017). Carriage of *sasX* has been shown to be vital to MRSA colonization and pathogenesis and was identified as a driver of MRSA epidemic waves (Li et al., 2012; Al Laham et al., 2015; Monecke et al., 2018) MRSA ST239 is currently not one of the major MRSA clones causing infections in Europe (Murray et al., 2022), but based on its infection-causing potential and its pandemic spread in Asia, our findings highlight the importance of continued genomic surveillance of MRSA in persons travelling to countries where MRSA is prevalent.

## Data availability statement

The datasets presented in this study can be found in online repositories. The names of the repository/repositories and accession number(s) can be found at: https://www.ncbi.nlm.nih.gov/genbank/, BioProject ID: PRJNA674016.

## Author contributions

JC, JL, AL, and SM-K: conceptualization. JC, BX, JV, and CL: experimentation. JC, BX, JV, and SP: bioinformatics and visualization. JC, JV, BX, JL, AL, and SM-K: manuscript writing and data interpretation. SM-K: supervision. All authors contributed to the article and approved the manuscript.

## Funding

JC was supported by the University of Antwerp doctoral assistant funds. BX was supported by University of Antwerp Research funds (BOF-DOCPRO 2012–27450).

## Conflict of interest

The authors declare that the research was conducted in the absence of any commercial or financial relationships that could be construed as a potential conflict of interest.

## Publisher’s note

All claims expressed in this article are solely those of the authors and do not necessarily represent those of their affiliated organizations, or those of the publisher, the editors and the reviewers. Any product that may be evaluated in this article, or claim that may be made by its manufacturer, is not guaranteed or endorsed by the publisher.

## Footnotes

^1^ www.danmap.org

^2^ http://phaster.ca/
